# Jumeaux conjoints thoracopages: à propos d’un cas à Libreville et revue de la littérature

**DOI:** 10.11604/pamj.2022.41.220.32282

**Published:** 2022-03-16

**Authors:** Aude Mariela Lembet Mikolo, Julienne Isabelle Minko, Eliane Kuissi Kamgaing, Mélina Marie Nkole Aboughe Obame, Angéla Mekame Méyé, Adryana Minouche Mabery Grodet Eyang, Live Gael Kiba, Daria Mindze Biyoghe, Simon Jonas Ategbo

**Affiliations:** 1 Centre Hospitalier Mère-Enfant, Fondation Jeanne Ebori, Libreville, Gabon,; 2 Département de Pédiatrie, Faculté de Médecine, Université des Sciences de la Santé, Libreville, Gabon,; 3 Centre Hospitalier Universitaire de Libreville, Libreville, Gabon,; 4 Département de Chirurgie Pédiatrique, Faculté de Médecine, Université des Sciences de la Santé, Libreville, Gabon

**Keywords:** Jumeaux conjoints, malformation, monochoriale, diagnostic anténatal, cas clinique, Conjoined twins, malformation, monochorionic, antenatal diagnosis, case report

## Abstract

Les jumeaux conjoints sont une malformation rare des grossesses gémellaires monozygotes et mono-amniotiques. Le pronostic fœtal de cette malformation reste très sombre, nécessitant un recours à une interruption de la grossesse si le diagnostic est posé précocement. Nous rapportons le cas de « jumeaux conjoints thoracopages » de H3 de vie, nés à terme à 40 SA + 3 jours, par voie basse. Ils sont nés de parents non consanguins, sans antécédent pathologique notable. La mère était jeune, primipare. La grossesse était suivie avec 5 consultations prénatales et 3 échographies obstétricales réalisées par un médecin inexpérimenté. Le diagnostic de jumeaux conjoints thoracopages a été évoqué à l´examen clinique et le bilan lésionnel établi à la tomodensitométrie (TDM). Cet examen a permis d´observer des anomalies cardiovasculaires incompatibles avec la vie. Aucune intervention n´a été faite, car les jumeaux décédaient à H23 de vie dans un tableau de détresse respiratoire aiguë. Les jumeaux conjoints constituent une anomalie congénitale rare. Un diagnostic échographique précoce permettrait de préciser les structures anatomiques communes, de rechercher une anomalie congénitale associée, d'organiser l'accouchement dans une structure adaptée et de programmer une prise en charge néonatale multidisciplinaire.

## Abstract (en)

Conjoined twins are a rare form of monozygotic and monoamniotic twins. Fetal prognosis is very poor and, if the diagnosis is made early, it most often requires therapeutic abortion. We here report a case of three-hour old thoracopagus conjoined twins who were born vaginally, at term, at 40 weeks + 3 days. They were born to non-consanguineous parents, without any significant pathological history. Their mother was young and primiparous. She received 5 antenatal visits and 3 obstetric ultrasounds were performed by an inexperienced physician. The diagnosis of thoracopages twins was suspected based on clinical examination and computerized tomography (CT) results. This test showed cardiovascular abnormalities incompatible with life. The twins died after 23 hours of age due to acute respiratory distress. Conjoined twins are a rare congenital abnormality. Early diagnosis based on ultrasound would help to determine common anatomical structures, investigate associated congenital abnormalities, schedule delivery in a suitable structure and multidisciplinary neonatal management.

## Introduction

Les jumeaux siamois sont l'une des rares anomalies congénitales, et parmi les plus grands défis de la chirurgie pédiatrique [1]. La prévalence des jumeaux conjoints est de 1/50000 à 200000 naissances, avec une prédominance féminine (70%) [2]. Environ 40 à 60% des cas sont morts nés et 35% survivent uniquement le premier jour [1, 2]. Les jumeaux siamois sont des jumeaux homozygotes soudés à la naissance. Actuellement, grâce à l'échographie, la plupart de ces grossesses sont dépistées précocement et une intervention thérapeutique peut être proposée. Le pronostic dépend du siège, de la nature, de l'extension des organes communs ainsi que de l'association à d'autres malformations [2]. Finalement, le sujet des jumeaux siamois ouvre un grand débat sur le plan éthique. Nous présentons le premier cas de jumeaux conjoints thoracopages à Libreville dont le diagnostic a été méconnu pendant la grossesse. À partir de cette observation et une brève discussion, nous ferons un rappel sur cette pathologie rarissime, sur son diagnostic échographique précoce pour établir une stratégie de prise en charge postnatale multidisciplinaire.

## Patient et observation

**Information du patient:** nouveau-nés de sexe masculin, nés par césarienne indiquée devant une grossesse gémellaire au terme de 40 semaines d´aménorrhée et 3 jours avec placentation monochoriale mono-amniotique. Ils ont été admis à H3 de vie pour prise en charge de jumeaux conjoints. Le bloc pesait 4160g. Le premier-né avait une taille de 42 cm et un périmètre crânien (PC) de 32 cm. Le deuxième né avait une taille de 43 cm et un PC de 33 cm. Ils sont nés en état de mort apparente avec un score d´Agar à 3/10 à la 1^re^ minute et 6/10 après une réanimation de 10 minutes selon l´algorithme de la réanimation. La mère était âgée de 16 ans, primipare, sans notion de maladies héréditaires. Le père était âgé de 17 ans. Les deux parents sont de nationalité Gabonaise, sans notion de consanguinité. La grossesse était suivie dans un centre de santé maternelle et infantile par 3 sages-femmes différentes. Elle a bénéficié de 5 consultations prénatales, toutes sans particularités. Les sérologies de la syphilis, des hépatites B et C, et du VIH étaient négatifs. Une immunité à la toxoplasmose et la rubéole était observée. Deux (2) échographies réalisées par un médecin généraliste, concluaient à une grossesse gémellaire mono-choriale, monoamniotique, évolutive XY avec une bonne vitalité sans aucunes anomalies. Elle avait reçu une supplémentation en fer et en acide folique.

**Figure 1:**
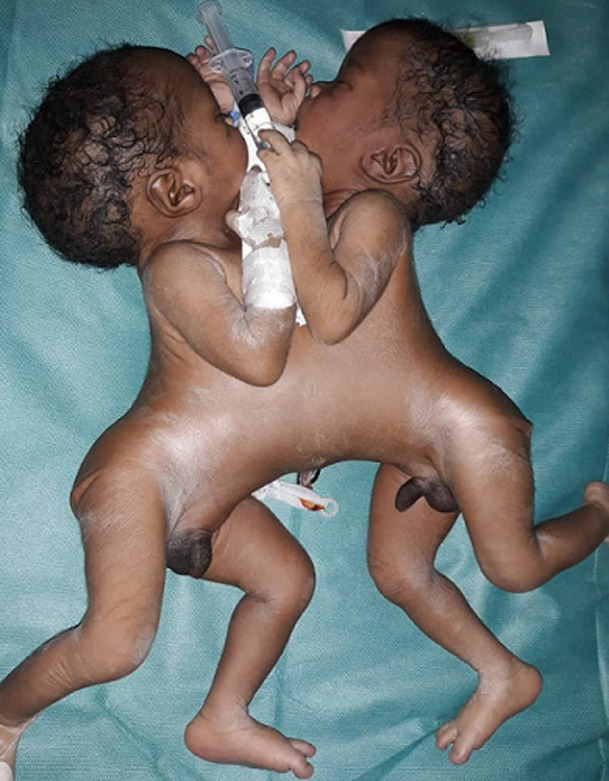
jumeaux conjoints thoracopage après extraction en post natal, vue d’ensemble

**Figure 2:**
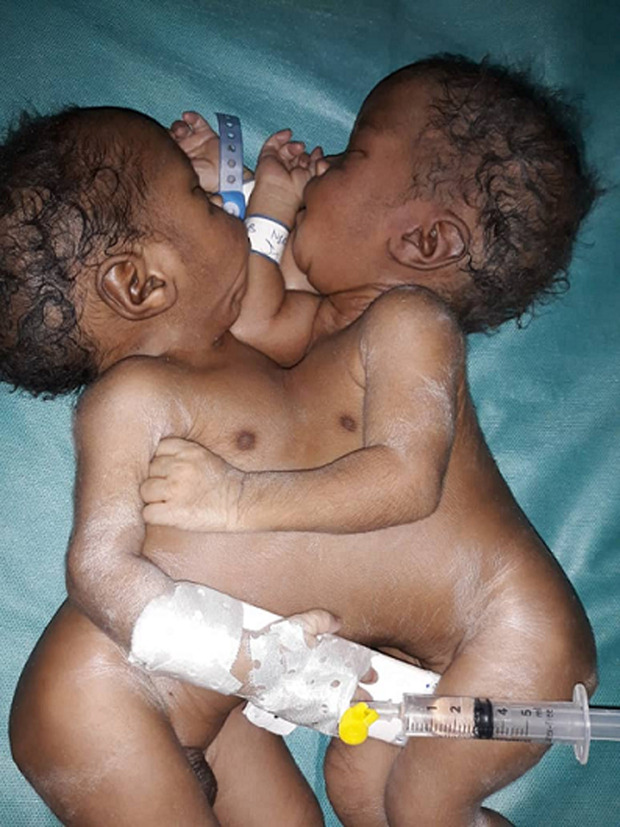
jumeaux conjoints thoracopage après extraction en post natal, vue antérieure

**Résultats cliniques:** l´examen à l´admission dans le service de médecine néonatale montrait des jumeaux conjoints fusionnés face à face de la partie supérieure du thorax à la partie supérieure de l´abdomen avec un abdomen difficile à examiner (Figure 1, Figure 2). La fréquence cardiaque était normale: 150 battements par minute (J1) et 132 battements par minute (J2). Ils présentaient une détresse respiratoire avec un score de Silverman à 6/10, une cyanose avec une saturation en oxygène de 82% a l´air ambiant et une polypnée à 70 cycles par minute. L´extrémité céphalique était de forme trigocéphale, les fontanelles larges, sutures normales, bouches et yeux sans particularités, oreilles bien ourlées (Figure 1, Figure 2). Les nouveau-nés étaient bien éveillés, toniques, réactifs, avec une bonne succion, les autres réflexes archaïques étaient difficiles à examiner. Les pouls périphériques étaient perçus. Les bruits du cœur audibles avec un rythme irrégulier et un souffle holosystolique. Les nouveau-nés présentaient chacun une tête et un cou complètements développés. Ils avaient une cage thoracique et une paroi abdominale supérieure communes. Chacun des jumeaux avait une paire de membres. Un seul cordon ombilical comprenant 6 vaisseaux (Figure 1, Figure 2). Un des jumeaux (J2) avait une imperforation anale. Le sexe était de type masculin, sans ambiguïté sexuelle avec une cryptorchidie droite de J2. L´absence d´autres malformations visibles était notée.

**Figure 3:**
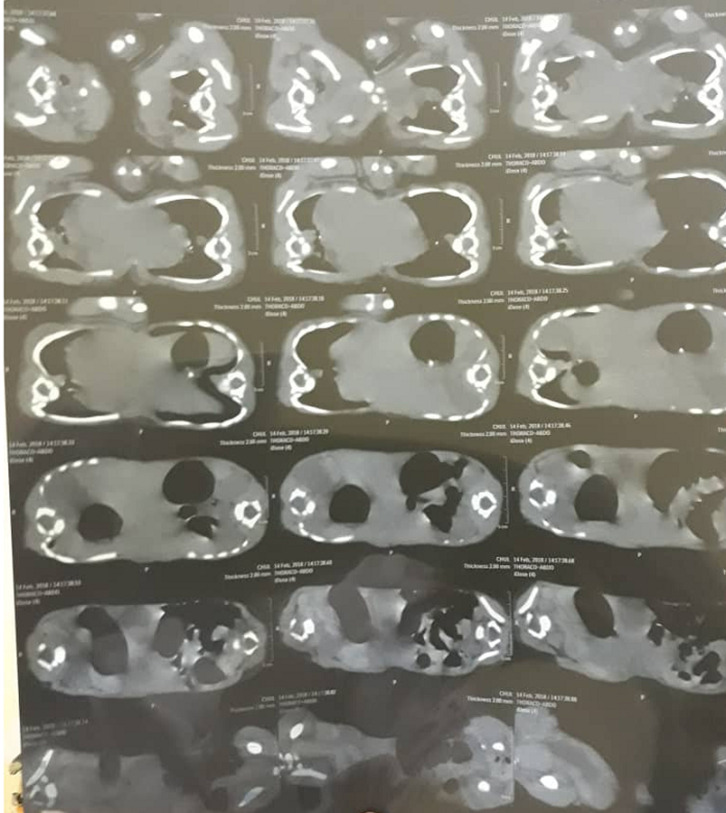
tomodensitométrie (étage thoracique) montrant un cœur unique

**Figure 4:**
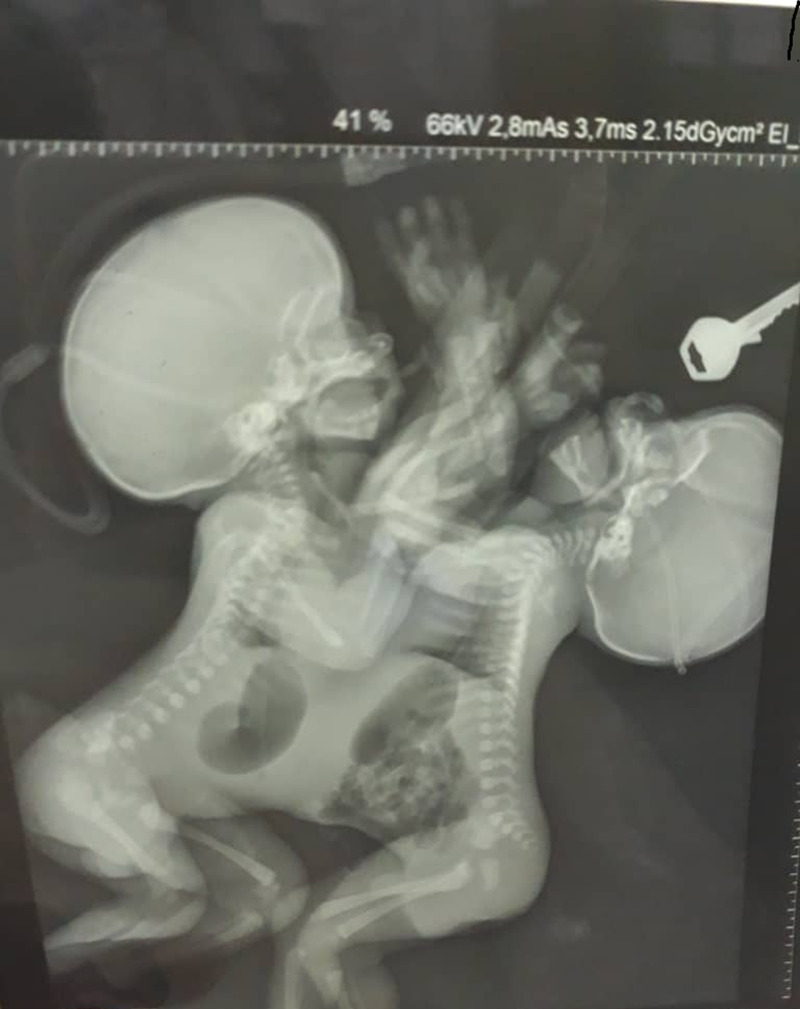
radiographie ASP (abdomen sans préparation) des jumeaux conjoints montrant des anses intestinales déviées à gauche

**Evaluation diagnostique:** les examens morphologiques ont mis en évidence au *Body-scanner:* une fusion du cœur, une voie unique, un sternum et un diaphragme commun, pas de cartilage chondro-costal antérieur, deux paires de reins distincts (Figure 3, Figure 4). L´étage cérébral était normal. Il n´y avait pas d´anomalies du squelette à la radiographie standard. L´abdomen sans préparation montrait des anses intestinales communes et deux estomacs (Figure 3). L´échographie abdominale montrait des jumeaux siamois partageant un foie avec kyste du foie et des reins individualisés). L´échographie cardiaque révélait deux cœurs fusionnés dont les 4 grandes cavités n´ont pas été distinctement individualisées. Ces 2 cœurs semblaient liés par une paroi commune. Le bilan biologique révélait: une altération de la fonction rénale avec l´urée (J1=3,40; J2=3,24mmol/L) et la créatinine (J1=278; J2=174umol/L). Une cytolyse hépatique avec les transaminases (J1= ASAT 354U/L, ALAT=50U/L; J2= ALAT=42, ASAT=205 UI/L), gamma GT (J1=41U/L; J2=68U/L), PAL (J1=25U/L; J2=56U/L). La glycémie normale (J1=4,55mmol/L; J2=4,92mmol). La protéine C réactive (CRP) négative et la calcémie était normale 2,3 mmol/l.

**Intervention thérapeutique et suivi:** le traitement initial consistait en une hospitalisation en soins intensifs, mise en incubateur ouvert, voie veineuse périphérique, mise en place d´une sonde orogastrique. Oxygénothérapie aux lunettes à 4l/minutes. Antibiothérapie probabiliste (Céfotaxime 50mg/kg/12h, Gentamycine 5mg/kg/j, Amoxicilline 50mg/kg/12h), restriction hydrique à 50ml/kg/j, gluconate de calcium à 60mg/kg/j. Malheureusement, l´évolution a été rapidement progressive et les nouveau-nés sont décédés à H23 de vie dans un tableau de détresse respiratoire aiguë sévère avec échec des mesures de réanimation. Aucune intervention chirurgicale n´a pu être faite.

## Discussion

Ce cas de jumeaux conjoints est le premier cas décrit à Libreville. L´absence d´intervention chirurgicale limite cette étude. Les jumeaux conjoints sont populairement connus sous le nom de jumeaux siamois, du nom du lieu de naissance des jumeaux siamois originels nés en 1811 au Siam (Thaïlande) [3]. Ainsi, la description des jumeaux siamois a été faite par les anciens égyptiens, mais le premier cas bien documenté était les servantes Bidden: Mary et Eliza Chulkhurst. Elles sont nées en 1100, à Kent, en Angleterre, unies par les hanches et les épaules. Après la mort de Mary à l´âge de 34 ans, une séparation immédiate a été refusée par Eliza qui a dit «comme nous sommes venues ensemble nous irons ensemble» et elle est décédé 6 heures après [1, 3]. Parmi également les jumeaux siamois, les plus célèbres sont Chang et Eng Bunker, nés au Siam (actuellement Thaïlande) en 1811 et ils sont décédés en 1874 à un âge de 63 ans. Il s´agissait de xiphopages unis à la partie inférieure du thorax. Ils sont devenus célèbres en travaillant dans un cirque international. Ils ont épousé deux sœurs et ont eu 21 enfants [1, 3]. Le premier diagnostic de jumeaux conjoints au cours d´une échographie réalisée sur une grossesse de 35 semaines en 1976 [1, 4]. Par la suite, les diagnostics sont faits de plus en plus précocement; même à sept semaines [1, 5]. La première opération de séparation réussie d´une paire de jumeaux siamois a été effectuée au XVIIe siècle par Johannes Fatio en Suisse. Il s'agissait de jumeaux unis par l'ombilic [1]. La dernière séparation concernait les sœurs siamoises Camerounaise Bissie et Eyenga qui étaient reliées par l´abdomen avec une partie du foie en commun. L´intervention avait eu lieu en France à Lyon le 13 novembre 2019 et avait durée sept heures. Les siamoises étaient âgées d´un an [6].

Les grossesses gémellaires monozygotes proviennent de la division précoce du même œuf. Il est décrit des jumeaux bichoriaux et biamniotiques (20 à 30%), lorsque la division survient dans les trois jours après la fécondation. Des jumeaux monozygotes, monochoriaux et biamniotique (70 à 80%), lorsque le clivage survient du 4^e^ au 8^e^ jour. Lorsque le clivage embryonnaire survient entre le 9^e^ et 13^e^ jour: les cellules de la lignée destinée à la formation du chorion et de l´amnios sont différenciées, tout clivage de l´œuf survenant à partir de ce moment aboutira à une grossesse gémellaire monochoriale monoamniotique. Ce sont les grossesses qui présentent plus de risque de complications. À partir du quatorzième jour après la fécondation, la division tardive et incomplète, aboutit à un monstre double ou jumeaux conjoints. Ce phénomène est très rare. Cependant, l´étiopathogénie des jumeaux conjoints est mal connue. Il n´y a pas d´anomalie chromosomique associée. La race, l´hérédité, la parité et la consanguinité n´interviendraient pas dans le processus. Deux théories opposées ont été proposés pour expliquer ce phénomène. Par ailleurs, la théorie de la fusion incomplète, tardive d´un seul embryon (théorie de la scission) est la plus acceptée [1, 3].

Plusieurs classifications ont été décrit en fonction du site d'union, des organes communs et de la symétrie. La classification de Spencer des siamois est universellement acceptée actuellement. Selon cette classification, on retrouve des jonctions dorsales (13%) à partir du tube neural ou ventral (87%) à partir de la ligne antérieure. Il en résulte huit types de duplication complète: les jumeaux conjoints céphalopages, thoracopages, omphalopages, ischiopages, parapages, craniopages, pygopages et rachipages [1, 3, 4]. À cette classification, il faut ajouter les duplications incomplètes et les formes rares: les diprosopes, les dicéphales, les dipygus, les jumeaux parasites et le fœtus in fœtus [5]. Il n'y a pas de cas connu de triplets ou de quadruplets conjoints [4]. Ce pendant, de nombreux cas de jumeaux conjoints dans une grossesse triple ont été décrits dans la littérature [7]. Nous avons retrouvé des cas de grossesse triple spontané ou par procréation médicale assistée monochoriale biamniotique [7, 8], des grossesses triples spontanées, bichoriale et biamniotique [9].

Le diagnostic anténatal du jumeau conjoint repose sur l'échographie. Le diagnostic précoce des jumeaux conjoints est possible au premier trimestre entre la 10^e^ - 13^e^ semaines d'aménorrhée (SA) [2, 4]. Certaines formes incomplètes peuvent être difficile à diagnostiquer, une échographie réalisée à 22 SA permet d'élucider ces formes, de localiser de manière plus précise la zone de rattachement, analyser les structures dupliquées et de chercher les malformations associées, en particulier les anomalies cardiaques [4]. Les autres techniques d'imagerie, à savoir l'imagerie par résonance magnétique du contenu utérin, imagerie 3D, sont utiles pour le diagnostic et peuvent apporter un complément d'information à visée pronostique [3, 4]. L´analyse précise du site d´union et des organes communs est améliorée par la voie trans-vaginale, voire par l´imagerie 3D et le Doppler [10]. L´accessibilité à l´échographie dans nos pays à faible densité médicale, ainsi que le coût de cet examen, rendent ce diagnostic tardif pour les non assurés à l´assurance maladie comme ce fut le cas pour cette parturiente qui a eu recours à des échographies réalisées par un médecin généraliste. Une échographie est souvent prescrite, mais pas toujours réalisée par un personnel qualifié. Une enquête sur les connaissances, attitudes et pratiques de l´échographie obstétricale au Sénégal, a révélé que 66,6% des praticiens (médecins et sages-femmes) n´étaient pas diplômés en échographie [5]. Ils étaient formés de façon empirique et présentaient d´importantes lacunes en termes de connaissance et de pratique de l´échographie.

Le plus souvent, le diagnostic échographique de jumeaux fusionnés conduit à l'interruption de grossesse [10]. Pour les jumeaux conjoints qui progressent vers l'accouchement, trois scénarios possibles existent: une séparation chirurgicale imminente, une séparation chirurgicale retardée ou aucune séparation. Lorsque la grossesse se poursuit jusqu'à l'accouchement, certaines situations exigent une opération en urgence lorsqu´un des jumeaux est mort-né ou en cas de mise en jeu du pronostic vital d´un des jumeaux par rapport à l´autre [1, 2]. D'autres interventions sont moins conséquentes lorsque aucun organe vital comme le cerveau ou le cœur n'est fusionné (dans ce cas, les vaisseaux sanguins nourrissent le corps des deux nouveau-nés). Lorsque la séparation est décidée après bilan et discussion, l'intervention chirurgicale se fera entre 6 et 12 mois de vie, laissant ainsi les nouveau-nés s'adapter et l´équipe multidisciplinaire de se préparer [1].

L'impression 3D est le terme utilisé pour la conception et la génération de modèles physiques. Les modèles 3D représentaient la taille exacte des organes vitale pour séparer et aidaient les chirurgiens à comprendre les relations spatiales. Actuellement, l'impression 3D joue 3 rôles majeurs, à savoir la planification chirurgicale, les simulations spécifiques aux patients et l'éducation. Une séparation chirurgicale complexe similaire d'une paire de jumeaux siamois connectés de la poitrine jusqu'au bassin a été réalisée avec succès par un groupe de chirurgiens du *Texas Children's Hospital de Houston* en 2015 à l'aide d'un modèle imprimé en 3D [3]. Le pronostic des jumeaux conjoints reste très réservé dans les pays en voie de développement. Leur survie dépend du type d´union (organes en commun) et des autres anomalies associées [2, 4]. Ce fut le cas dans notre observation où les jumeaux conjoints avaient en commun le cœur et le foie. L'opération chirurgicale pour séparer des siamois peut être très complexe. Il s'agit d'une chirurgie lourde, difficile, longue et risquée pour les patients. La mortalité néonatale des jumeaux conjoints est généralement élevée [1].

## Conclusion

Considéré comme des monstres, les jumeaux conjoints constituent une anomalie congénitale rare. Le diagnostic repose sur l´échographie qui doit être réalisée par des obstétriciens expérimentés. Aussi, un diagnostic anténatal précoce permettrait de préciser les structures anatomiques communes, de rechercher une anomalie congénitale associée, d'organiser l'accouchement dans une structure adaptée et de programmer une prise en charge néonatale. À l´ère de l´échographie, aucun diagnostic de jumeaux conjoints ne devrait être une surprise de l´accouchement, même dans les pays en développement. La séparation réussie de jumeaux siamois nécessite l'effort conjoint d´une équipe multidisciplinaire expérimentée pour une meilleure prise en charge dans les pays en voie de développement. Finalement, notre sujet est l'une des rares anomalies congénitales, mais parmi les plus grands défis de la chirurgie pédiatrique moderne et un sujet d´intérêt.
